# Solubility Enhancement of Myricetin by Inclusion Complexation with Heptakis-*O*-(2-Hydroxypropyl)-β-Cyclodextrin: A Joint Experimental and Theoretical Study

**DOI:** 10.3390/ijms21030766

**Published:** 2020-01-24

**Authors:** Dongxu Han, Zhongbao Han, Liyan Liu, Ying Wang, Shigang Xin, Hongbo Zhang, Zhan Yu

**Affiliations:** 1 School of Chemistry and Chemical Engineering, Shenyang Normal University, Shenyang 110034, China; 2 Experimental Center, Shenyang Normal University, Shenyang 110034, China

**Keywords:** myricetin, HP-β-CD, phase solubility, molecular dynamics, PM6-D3H4, PM7

## Abstract

Four cyclodextrins (CD) including β-cyclodextrin (β-CD), γ-cyclodextrin (γ-CD), heptakis-*O*-(2-hydroxypropyl)-β-cyclodextrin (HP-β-CD), and heptakis-*O*-(2, 6-di-*O*-methyl)-β-cyclodextrin (DM-β-CD) were used as solubilizer to study the solubility enhancement of myricetin. The results of the phase solubility study showed that the presence of CDs could enhance the solubility of myricetin by forming 1:1 complexes. Among all CDs, HP-β-CD had the highest solubilization effect to myricetin. The concentration of myricetin could be 1.60 × 10^−4^ moL/L when the presence of HP-β-CD reached 1.00 × 10^−2^ moL/L, which was 31.45 times higher than myricetin’s aqueous solubility. Subsequently, the HP-β-CD:myricetin complex was characterized by Fourier transform infrared spectroscopy (FT-IR), X-ray diffraction (XRD), and thermogravimetric analysis (TGA). In order to get an insight of the plausible structure of the complex, molecular docking was used to study the complexation process of HP-β-CD and myricetin. In the complex, the A ring and C ring of myricetin were complexed into the hydrophobic cavity of HP-β-CD, while the ring B was located at the wide rim of HP-β-CD. Four hydrogen bonding interactions were found between HP-β-CD and -OH groups of the guest in the HP-β-CD: myricetin complex. The complexation energy (△*E*) for the host-guest interactions was calculated with a negative sign, indicating the formation of the complex was an exergonic process. A 30-ns molecular dynamics simulation was conducted to the HP-β-CD: myricetin complex. Calculation results showed that no large structural deformation or position change were observed during the whole simulation time span. The average root-mean-square deviation (RMSD) changes of the host and guest were 2.444 and 1.145 Å, respectively, indicating the complex had excellent stability.

## 1. Introduction

Cyclodextrins (CDs) [1,2] are natural cyclic oligosaccharides. CDs containing six, seven, and eight D-(+)-glucopyranose units linked by α-1,4 glycosidic bonds are named as α-CD, β-CD, and γ-CD [3], respectively. Owing to the lack of free rotation about chemical bonds connecting the glucopyranose units, CDs are not perfectly cylindrical molecules but are toroidal or cone shaped [4], with hydrophilic hydroxyl groups on their outer surface and a hydrophobic cavity in the center. The unique structure enables CDs to form non-covalent complexes, encapsulating either the entire or part of the guest molecule inside its cavity. The molecular inclusion complex formed by CDs manifests a profound effect on the solubility, stability, and physicochemical properties of the guest molecule [5]. Due to the presence of intramolecular hydrogen bonding, natural CDs have remarkably low water solubility, which limits their practical application. In order to overcome this shortcoming, chemically derived CDs have been synthesized such as heptakis-*O*-(2, 6-di-*O*-methyl)-β-cyclodextrin (DM-β-CD), and heptakis-*O*-(2-hydroxypropyl)-β-cyclodextrin (HP-β-CD). CD derivatives and their inclusion complexes are widely used in the food, pharmaceutical, and cosmetic industries [6].

**Figure 1:**
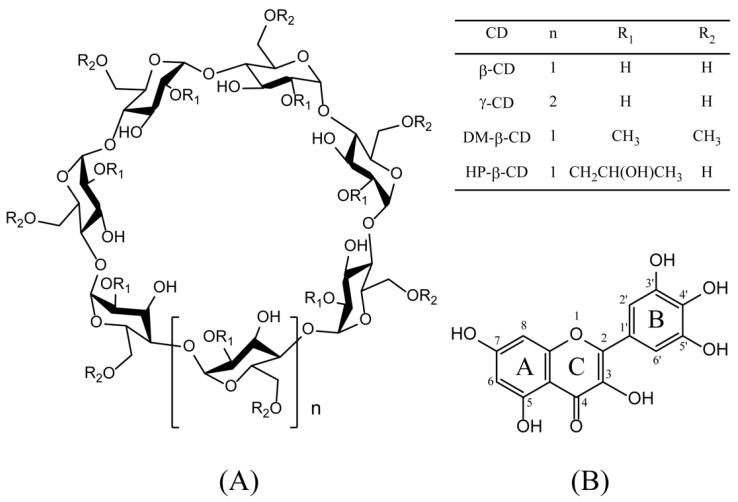
Chemical structures of cyclodextrins (CDs) (**A**) and myricetin (**B**). Abbreviations: β-cyclodextrin, (β-CD); γ-cyclodextrin, (γ-CD); heptakis-*O*-(2-hydroxypropyl)-β-cyclodextrin, (HP-β-CD); and heptakis-*O*-(2, 6-di-*O*-methyl)-β-cyclodextrin, (DM-β-CD).

Myricetin, a natural flavonol with hydroxyl groups at 3, 5, 7, 3’, 4’, and 5’ positions (Figure 1), exists in the bark and leaves of *Myrica rubra*. Myricetin is often used as traditional medicine to control glycemia of diabetic patients in northern Brazil [7,8]. Currently, this compound can be used as a flavor modifier in snack foods, dairy products, and beverages in Japan [7,9,10]. Recent studies have also indicated that myricetin has potent antioxidant, anti-inflammatory, and anti-tumor functions [11,12]. However, myricetin is nearly insoluble in water [13,14], leading to low bioavailability. Thus, myricetin is usually administered orally as aqueous suspensions, reducing its clinical applications [15].

The aim of this study is to probe whether the aqueous solubility of myricetin can be successfully increased by the formation of a CD: myricetin complex. Four CDs, including β-CD, γ-CD, HP-β-CD, and DM-β-CD, were used as solubilizers in this work. The results of this study indicated that HP-β-CD is the most potent solubilizer for myricetin. UV-Vis spectroscopy, IR spectroscopy, X-ray diffraction (XRD), and thermogravimetric analysis (TGA) were used to characterize the inclusion complex of HP-β-CD: myricetin. Molecular docking was used to investigate the plausible structure of HP-β-CD: myricetin complex. A 30-ns molecular dynamic simulation was also applied to the lowest energy docking result to investigate the stability of the complex.

## 2. Results and Discussions

### 2.1. Phase Solubility Study

**Figure 2:**
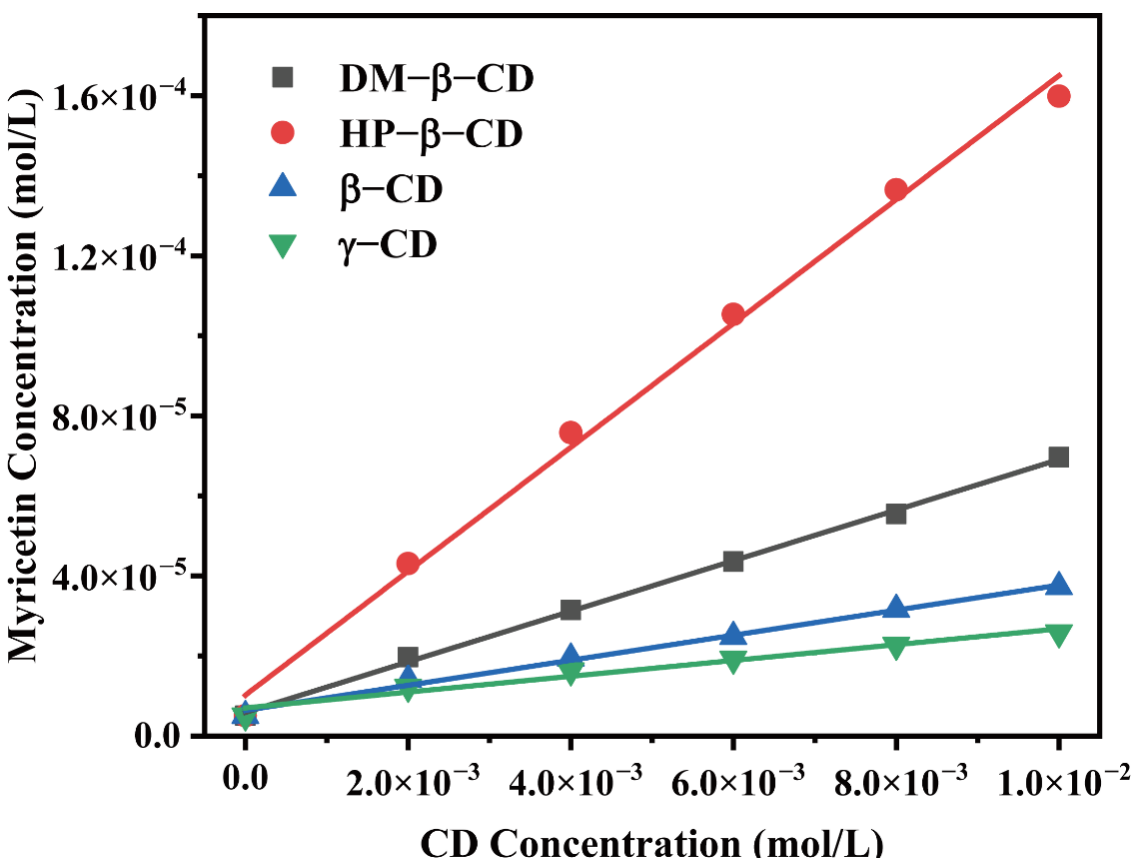
Phase solubility diagrams of myricetin in aqueous solution with the presence of various CDs.

The phase solubility diagram was obtained by measuring the molar concentration of the myricetin in the presence of four CDs at various concentrations, as shown in Figure 2. The solubility of myricetin linearly increased as a function of CD concentration, as CD concentrations increased from 0.0 to 1.0 × 10^−2^ moL/L. The enhancement in the solubility of myricetin confirmed intermolecular interactions existed between the host and the guest. Furthermore, the profile of all diagrams could be classified as A_L_ type according to the method reported by Higuchi and Conners [16], indicating 1:1 molecular complexes formation.

The apparent stability constant (*K*) of the host–guest complex could be an indication for probing the binding strength between the host and the guest molecule. *K* values of CD: myricetin complexes (1:1) were calculated from the linear plots of the phase solubility diagram and shown in Table 1. Among four CD: myricetin complexes, HP-β-CD: myricetin had a *K* value of 3090.48 L/mol, which is higher than that reported by Yao, et al. [17], where a suspension method was adopted to get the HP-β-CD: myricetin complex. Myricetin solubility could increase 31.45-fold when HP-β-CD was used in the concentration of 1.00 × 10^−2^ moL/L.

### 2.2. Fourier Transform Infrared Spectroscopy

**Figure 3:**
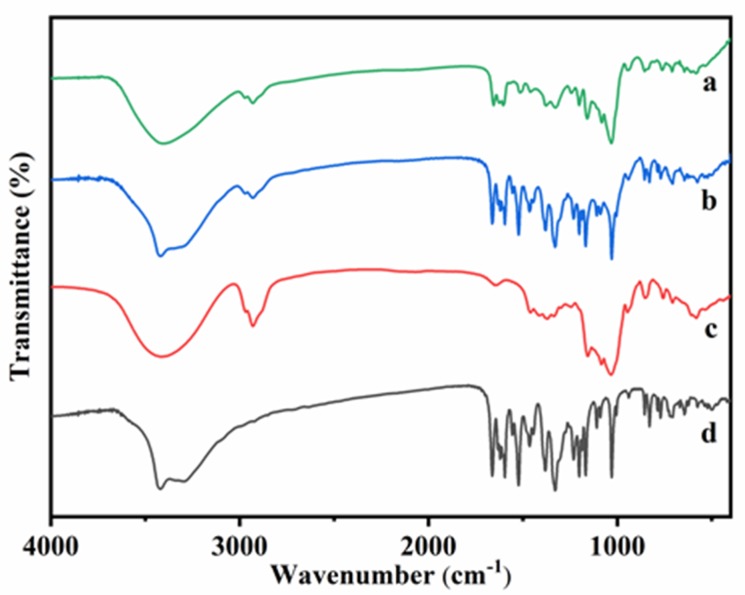
Fourier transform infrared spectroscopy (FT-IR) spectra of HP-β-CD: myricetin inclusion complex (**a**), HP-β-CD: myricetin physical mixture (**b**), HP-β-CD (**c**) and myricetin (**d**).

The Fourier transform infrared spectroscopy (FT-IR) spectra of HP-β-CD: myricetin complex, HP-β-CD: myricetin physical mixture, HP-β-CD, and myricetin were presented in Figure 3. The FT-IR spectrum of HP-β-CD showed prominent absorption bands at 3415 cm^−1^ (O-H stretching), 2929 cm^−1^ (C-H stretching), 1645 cm^−1^ (H-O-H bending), 1157 cm^−1^ (C-O stretching), and 1031 cm^−1^ (C-O-C stretching), which were similar to the results obtained by Gidwani, et al. [18]. The FT-IR spectrum of myricetin showed characteristics bands at 1662 cm−1 (C-C stretching of A ring), 1594 cm^−1^ (C=O stretching), 1521 cm^−1^ (C=C stretching of A ring), 1380 cm^−1^ (C–C stretching), 1328 cm^−1^ (C-C stretching of B ring), and 1030 cm^−1^ (C-O stretching of B ring), which were almost consistent with the IR spectrum of myricetin [19].

In the spectrum of physical mixture, almost characteristic bonds of both HP-β-CD and myricetin were evident (3420, 2929, 1662, 1594, 1521, 1380, 1328, and 1030 cm^−1^), which indicated that there was almost no interaction between HP-β-CD and myricetin when the two compounds were physically mixed. However, in the HP-β-CD: myricetin inclusion complex, absorption bands of myricetin at 1662, 1594, and 1521 shifted to 1654, 1602, and 1512, respectively, indicating that the aromatic A ring, O–H and C = O groups of myricetin were likely involved in the interactions with HP-β-CD.

### 2.3. X-Ray Diffraction (XRD)

**Figure 4:**
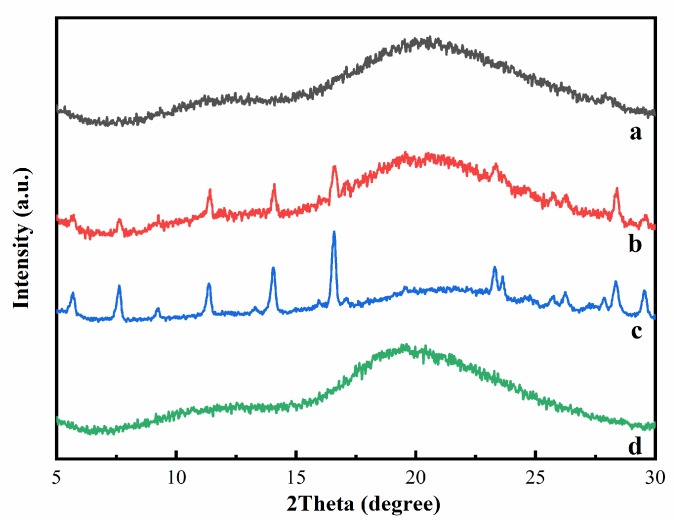
X-ray diffraction (XRD) patterns of HP-β-CD: myricetin inclusion complex (**a**), HP-β-CD: myricetin physical mixture (**b**), myricetin (**c**), and HP-β-CD (**d**).

Figure 4 presented the XRD patterns of HP-β-CD, myricetin, their inclusion complex, and physical mixture. For HP-β-CD, only one broad peak centered at 19.5° was observed, consistent with its amorphous nature. The XRD patterns of myricetin displayed some sharp peaks at 5.68, 7.62, 9.22, 11.36, 14.06, 16.58, 23.32, 23.62, 28.38, and 29.54°, indicating the crystallinity characteristic of this compound. The XRD of the physical mixture of HP-β-CD and myricetin was a superposition of the patterns of the components, confirming there was no chemical interactions between HP-β-CD and myricetin, and both kept their original physical characteristics. On the contrary, the XRD patterns of the HP-β-CD: myricetin complex (curve a in Figure 4) exhibited typical halo patterns for its amorphous structure [20], showing myricetin might be fully included in the cavity of HP-β-CD [21].

### 2.4. Thermogravimetric Analysis (TGA)

**Figure 5:**
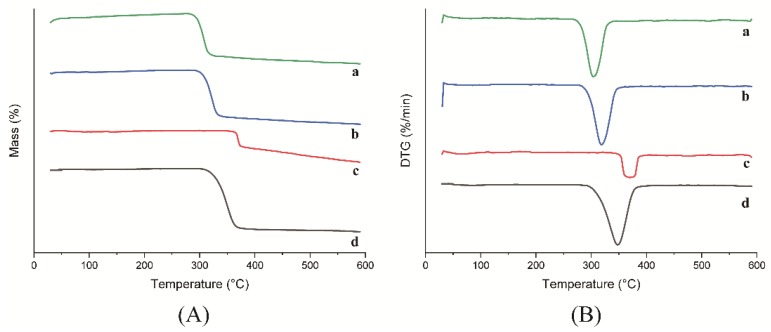
TG (**A**) and DTG (**B**) curves of HP-β-CD: myricetin inclusion complex (a), HP-β-CD: myricetin physical mixture (b), myricetin (c) and HP-β-CD (d).

The thermogravimetry (TG) and derivative thermogravimetry (DTG) curves for HP-β-CD: myricetin inclusion complex, physical mixture, HP-β-CD, and myricetin are shown in Figure 5. HP-β-CD presented almost no mass loss within the temperature range of room temperature to 300 °C. In addition, HP-β-CD lost 86.86% of its mass in the temperature range of 300–400 °C, with a maximum degradation rate at 347.6 °C. Furthermore, HP-β-CD lost 3.44% of its mass in the temperature range of 400–590 °C. Myricetin displayed a different degradation process of losing 26.08% and 19.19% of its mass in the temperature range of 300–400 °C and 400–590 °C, respectively, with a maximum mass variation rate at 369.9 °C, which is similar to other reports [22].

The HP-β-CD: myricetin inclusion complex showed a maximum mass loss rate at 302.65 °C lower than that of the physical mixture at 317.83 °C. The difference in melting point indicated a decrease of thermal stability of the HP-β-CD: myricetin complex, which was the consequence of the guest amorphization through the formation of the inclusion complex [23]. Similar results were also reported for the inclusion process of other substances [24,25].

### 2.5. Molecular Modeling Studies

**Figure 6:**
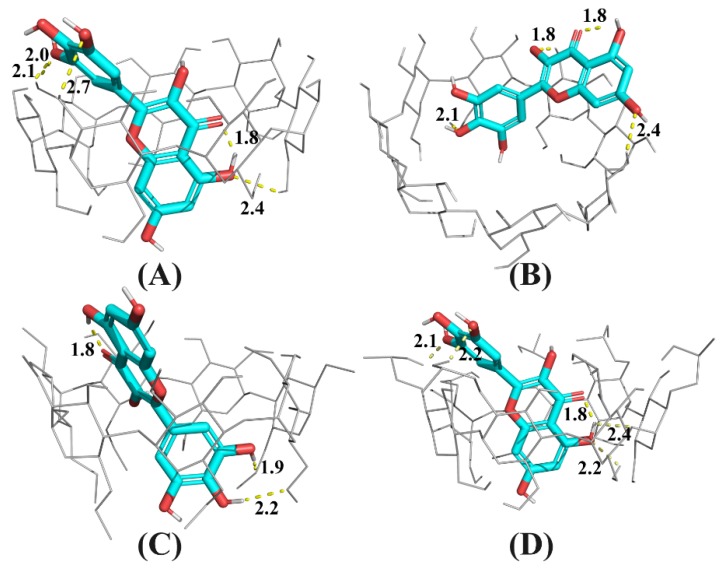
Snapshot of the lowest energy docking result for CD: myricetin complexes, where CD is β-CD (**A**), γ-CD (**B**) DM-β-CD (**C**), or HP-β-CD (**D**). Hydrogen bonds are dashed lines with indicated distances (in Å).

In this work, a molecular docking technique was applied to probe the formation process of the CD: myricetin complex. After 300 runs of molecular docking, only the complex structure in the lowest energy result was considered as the top-docking, shown in Figure 6.

As shown in Figure 6B, because the cavity size of γ-CD was 2.5-fold larger than that of β-CD [26] and its derivatives, myricetin was docked at the secondary rim of γ-CD, with the B ring intruding the cavity of γ-CD. For β-CD and its derivatives, myricetin was largely accommodated by their more shape- and size-fit cavities. The A and C ring of myricetin was included inside cavities of β-CD and HP-β-CD, but the B ring was included by DM-β-CD. Because DM-β-CD has a reduced cavity volume compared with natural β-CD [26], the top-docking results seems plausible.

As shown in Figure 6D, the A and C ring of myricetin was accommodated by HP-β-CD cavity and the B ring located at the secondary rim of HP-β-CD. There existed an intramolecular hydrogen bond between the carbonyl group at C4 and the hydroxyl group at C5, in accordance with the finding by Vojta, et al. [27]. Additionally, it can be found that there are several hydrogen bonds between the host and guest in Figure 6D, indicating hydrogen bonding as well as hydrophobic interactions were driving forces for the formation of the HP-β-CD: myricetin complex.

The complexation energy (Δ*E*) of complex and the isolated molecules could be considered for evaluation of the stability of the complex. To this purpose, the binding energy of HP-β-CD: myricetin complex was evaluated quantum mechanically at a semi-empirical level using the PM6-D3H4 and PM7 method.

PM6-D3H4 [28] introduces dispersion and hydrogen-bonded corrections to the PM6 method and PM7 is the latest parametrization method for MOPAC. Table 2 gives the calculation results by using the PM6-D3H4 and PM7 method. Both methods give negative results of △*E* in the same scale; namely, the inclusion complex has lower energy (heat) of formation (*E*) than the sum of the heat of formation of the isolated host and guest molecule, representing the formation of the complex was thermodynamically spontaneous.

**Figure 7:**
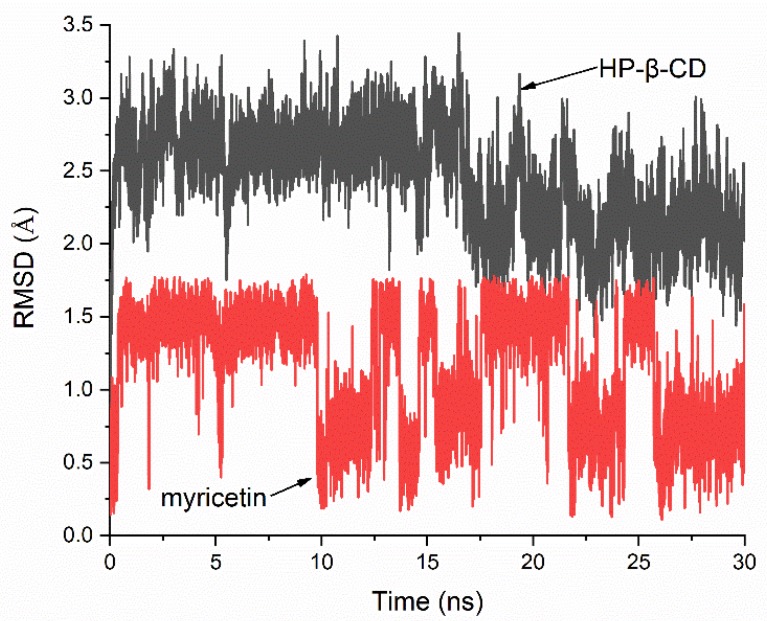
Root-mean-square deviation (RMSD) plots of all atoms for HP-β-CD: myricetin complex.

The stability of the HP-β-CD: myricetin complex was evaluated by a 30-ns molecular dynamics (MD) simulation of the lowest energy molecular docking result. Figure 7 shows the root-mean-square deviation (RMSD) value of the host and guest relative to their initial positions. It can be seen that when the MD simulation began, the system quickly reached equilibrium and the changes in atomic positions in both the host and guest were in a very small range. The mean RMSD of HP-β-CD and myricetin were 2.444 Å and 1.145 Å, respectively, and the standard deviation (SD) were 0.362 Å and 0.412 Å, indicating the host and guest had almost no large positional change and structural deformation. The MD simulation result supports that the structure of the lowest energy molecular docking result had enough stability.

## 3. Materials and Methods

### 3.1. Instrumentation

UV-Vis spectra were recorded on a UH 5300 UV-vis spectrometer (Hitachi, Tokyo, Japan) at room temperature. Data were collected in the range of 200–800 nm, with the band width of 1 nm and with the data interval of 0.5 nm.

IR spectra were collected on a Nicolet 380 Fourier transform infrared spectrometer (Thermal Scientific, Waltham, MA, USA). Spectra were collected in the 4000−400 cm^−1^ spectral range, with a resolution of 2 cm^−1^ and with 32 acquisitions as co-added scans.

XRD data were obtained by using an Ultima IV X-ray powder diffractometer (Rigaku, Tokyo, Japan) with Cu-Kα irradiation (λ = 1.5418 Å). The operation conditions of analysis were a voltage of 40 kV, current of 40 mA, scanning range 5–30°, and scan rate of 0.02° min^−1^.

Thermogravimetric analysis was applied on a STA449 F5 thermal analyzer (NETZSCH, Selb/Bavaria, Germany). Samples 2.0 to 2.5 mg were heated from room temperature to 590 °C at the heat rate of 10 °C min^−1^.

### 3.2. Materials

Myricetin was purchased from Dasfu Biological Company (Nanjing, China); β-CD, γ-CD, HP-β-CD, and DM-β-CD were purchased from Yuanye Biotechnology (Shanghai, China). Other chemicals and solvents used in this study are of analytical grade or higher. Ultrapure water (18.2 MΩ·cm) was used in solution preparation and dilution.

### 3.3. Phase Solubility Study

Phase solubility study was performed according to Higuchi and Connors’ method [16]. An excess amount of myricetin (1.0 mg) was added to disposable centrifuge tubes containing 5.0 mL of distilled water with various concentrations of CDs (0–1.0 × 10^−2^ moL/L). These tubes were sonicated continuously for 30 min to reach equilibrium and then centrifuged at 4000 r/min for 20 min at room temperature. Supernatant were collected and assayed at 378  nm. Each measurement was run in triplicate.

According to Higuchi and Connors’ method, the stability constant (K) of the solute and the solubilizer can be calculated from the phase solubility diagram using the equation of

(1) $$K=\frac{\mathrm{slope}}{s_{0}\left(1-\mathrm{slope}\right)}$$

where slope is obtained from the plot of myricetin concentration versus CD concentration and s_0_ is the aqueous solubility of myricetin. Based on preliminary experiments, s_0_ of myricetin is found of 5.088 × 10^−6^ moL/L^−1^.

### 3.4. Preparation of HP-β-CD: Myricetin Inclusion Complex and Physical Mixture

The physical mixture was prepared by simple blending HP-β-CD and myricetin in a mortar with pestle until a homogeneous mixture was found [29].

HP-β-CD: myricetin inclusion complex were prepared by using the kneading method described by Doile, et al. [23] with certain modifications. First, several drops of water were added to the HP-β-CD powder placed in a mortar. After kneading for several minutes, a homogeneous paste was obtained. Then, myricetin was carefully added to the paste with a small amount of ethanol. The mixture was kneaded for more than 45 min and dried at 50 °C for 24 h.

### 3.5. Molecular Modeling, Docking, and Dynamics Studies

The three-dimensional structure data of β-CD and γ-CD were taken from the Cambridge Crystal Library (CCDC) with entries of ARUXIU and CYDXPL, respectively. HP-β-CD and DM-β-CD were generated based on the β-CD structure by PyMol. The 3D structure of myricetin was taken from PubChem (https://pubchem.ncbi.nlm.nih.gov). All structures were subjected to structural optimization pretreatment by MMFF94 [30] force field before performing molecular simulation calculations. Autodock 4.2 (Scripps Research Institute, San Diego, CA, USA) [31] was used to study the intermolecular interactions between the host and the guest. Rotatable single bonds and non-polar hydrogen atoms of the guest were kept according to the default parameters of Autodock. All bond flexible rotations of the guest were allowed. Lamarckian genetic algorithm (LGA) was selected in this study. Some important parameters for LGA were: 300 for GA runs, 2,500,000 for energy evaluations, and 150 for LGA population size.

The lowest energy conformation of docking simulation was extracted and analyzed. MOPAC 2016 program (Stewart Computational Chemistry, Colorado Springs, CO, USA) [32] was used to calculate the complexation energy (△*E*). Both PM6-D3H4 and PM7 force fields were adopted. Other keywords for MOPAC calculations were: EF PRECISE CHARGE = 0 GNORM = 0.1 XYZ THERMO (298,298) LET. The complexation energy (Δ*E*) was defined as the difference between the heat of complex formation and the heat of involved free molecules represented by the following equation [33]:

(2) $$△E=E_{complex}-E_{host}-E_{guest}$$

All molecular dynamics simulations were carried out with Desmond 2018.4 (DE Shaw Research, NY, USA) [34]. The lowest energy conformation of docking was introduced into a cubic box with a side length of 10 Å, which was filled with TIP3P model water molecules. The default relaxation protocol was applied. Production runs were performed in the NPT ensemble at 300 K and 1.013 bar for 30 ns. No buffer ions were added into the system. The temperature and pressure were controlled using a Nose-Hoover thermostat (relaxation time 1.0 ps) and a Martyna-Tobias-Klein barostat (relaxation time 2.0 ps). The OPLS3 force field was used during the MD simulations.

## 4. Conclusions

In this work, the phase solubility method was used to compare the solubilization effect of β-CD, γ-CD, DM-β-CD, and HP-β-CD to myricetin. Experimental results show that HP-β-CD has the highest solubilization effect to myricetin by forming a 1:1 complex. The existence of HP-β-CD: myricetin complex was also characterized by FT-IR, XRD, and TG techniques. The plausible structure of the complex was probed by molecular docking. Semi-empirical quantum calculation and MD simulation applied to the best docking result testified the thermodynamic and stability of the structure of the complex. The results of this study highlight HP-β-CD is a good solubilizer for insoluble molecules. HP-β-CD might have great potent in industries of herbal medicines or healthcare products due to its relatively high-water solubility with low toxicity.

## Figures and Tables

**Table 1:** The Linear regression equation and the stability constants for CD: myricetin inclusion complexes.

CD	Linear Regression Function	*R* ^2^	*K* (L/mol)
DM-β-CD	Y = 0.00632c + 5.90344 × 10^−6^	0.9984	1250.11
HP-β-CD	Y = 0.01548c + 1.02057 × 10^−5^	0.9940	3090.48
β-CD	Y = 0.00313c + 6.42576 × 10^−6^	0.9920	617.14
γ-CD	Y = 0.00198c + 7.0336 × 10^−6^	0.9629	389.95

**Table 2:** Final heat of formation energy (*E*) and the complexation energy (∆*E*) for the HP-β-CD: myricetin complex from semi-empirical quantum mechanical PM6 and PM7 methods.

Method	*E*_complex_ (kcal/mol)	*E*_host_ (kcal/mol)	*E*_guest_ (kcal/mol)	△*E* (kcal/mol)
PM6-D3H4	−1820.803	−1530.570	−259.984	−30.249
PM7	−1847.438	−1548.061	−258.161	−41.216
